# Biclique: an R package for maximal biclique enumeration in bipartite graphs

**DOI:** 10.1186/s13104-020-04955-0

**Published:** 2020-02-21

**Authors:** Yuping Lu, Charles A. Phillips, Michael A. Langston

**Affiliations:** 1 Lawrence Berkeley National Laboratory, Berkeley, CA 94720 USA; 2 Department of Electrical Engineering and Computer Science, University of Tennessee, Knoxville, TN 37996-2250 USA

**Keywords:** Biclique, Bipartite graph, Graph algorithms, Maximality, R package

## Abstract

**Objective:**

Bipartite graphs are widely used to model relationships between pairs of heterogeneous data types. Maximal bicliques are foundational structures in such graphs, and their enumeration is an important task in systems biology, epidemiology and many other problem domains. Thus, there is a need for an efficient, general purpose, publicly available tool to enumerate maximal bicliques in bipartite graphs. The statistical programming language R is a logical choice for such a tool, but until now no R package has existed for this purpose. Our objective is to provide such a package, so that the research community can more easily perform this computationally demanding task.

**Results:**

*Biclique* is an R package that takes as input a bipartite graph and produces a listing of all maximal bicliques in this graph. Input and output formats are straightforward, with examples provided both in this paper and in the package documentation. *Biclique* employs a state-of-the-art algorithm previously developed for basic research in functional genomics. This package, along with its source code and reference manual, are freely available from the CRAN public repository at https://cran.r-project.org/web/packages/biclique/index.html.

## Introduction

All graphs we consider are finite, simple, unweighted and undirected. They are also *bipartite*, which means their vertices can be partitioned into two *partite sets* so that the endpoints of each edge lie in different sets. In such a graph, a *biclique* is a complete bipartite subgraph, that is, a subgraph in which every subgraph vertex in one partite set is adjacent to every subgraph vertex in the other partite set. A biclique with *p* vertices in one partite set and *q* vertices in the other is denoted by *K*_*p,q*_. A biclique is *maximum* if it is of largest size, with size measured by either its number of vertices (vertex-maximum) or its number of edges (edge-maximum). Finding a vertex-maximum biclique is *NP*-hard [1], while identifying an edge-maximum biclique can be accomplished in polynomial time [2]. A biclique is *maximal* if no vertex can be added to it to form a larger biclique.

The problem of enumerating all maximal bicliques has found utility in a host of applications. In the biological sciences, for example, it has been used for biclustering microarray data [3–5], modeling proteome-transcriptome relationships [6], identifying discriminating genotype patterns [7], optimizing phylogenetic tree reconstructions [8], discovering epidemiological patterns [9], identifying common gene-set associations [10], and integrating heterogeneous functional genomics data [11]. This problem is difficult in large part due to its combinatorial nature. A bipartite graph with *n* vertices may contain as many as *2*^*n/2*^ maximal bicliques [12].

In previous work [13], we presented a fast, general-purpose algorithm for this task. We dubbed it the Maximal Biclique Enumeration Algorithm, MBEA, and presented along with it an improved version we termed iMBEA. In this paper, we describe a publicly available implementation of both algorithms wrapped in R [14]. Simply called *Biclique*, this R package invokes efficient implementations of MBEA and iMBEA written in C. Our goal is to provide the scientific community with a practical, convenient and efficient tool for finding all maximal bicliques in bipartite graphs.

## Main text

### Implementation

*Biclique* consists of four R functions. The core function, *bi.clique*, invokes an efficient algorithm to enumerate maximal bicliques. Three utility functions, *bi.format*, *bi.print*, and *bi.degree*, provide formatting and output support.

The *bi.clique* function takes five arguments, four of which have default values. These five are: an input file name, an input file format (either an edge list (the default) or a binary matrix), two arguments, one for each partite set, that specify the minimum number of vertices required for a maximal biclique to be reported (the default is 3), and an argument specifying the algorithm to use, either MBEA or iMBEA (the default is iMBEA). Pseudocode for MBEA and iMBEA is shown in Algorithm 1. Because iMBEA differs from MBEA by only a handful of additional steps, the two algorithms are presented jointly, with starred lines denoting the steps unique to iMBEA. On dense graphs, iMBEA will usually be the faster algorithm, while on sparse graphs, both algorithms are apt to take about the same amount of time. We therefore recommend the use of iMBEA in most cases. See [13] for a thorough discussion of the two methods.

The three utility functions operate as follows. The *bi.print* function generates a visual histogram of the distribution of sizes of the maximal bicliques enumerated by the most recent call to *bi.clique*. The *bi.format* function augments a list of edges with a header line declaring the number of vertices and edges the list contains, as is required by *bi.clique*. The *bi.degree* function reads a bipartite graph and outputs the degree of each vertex.
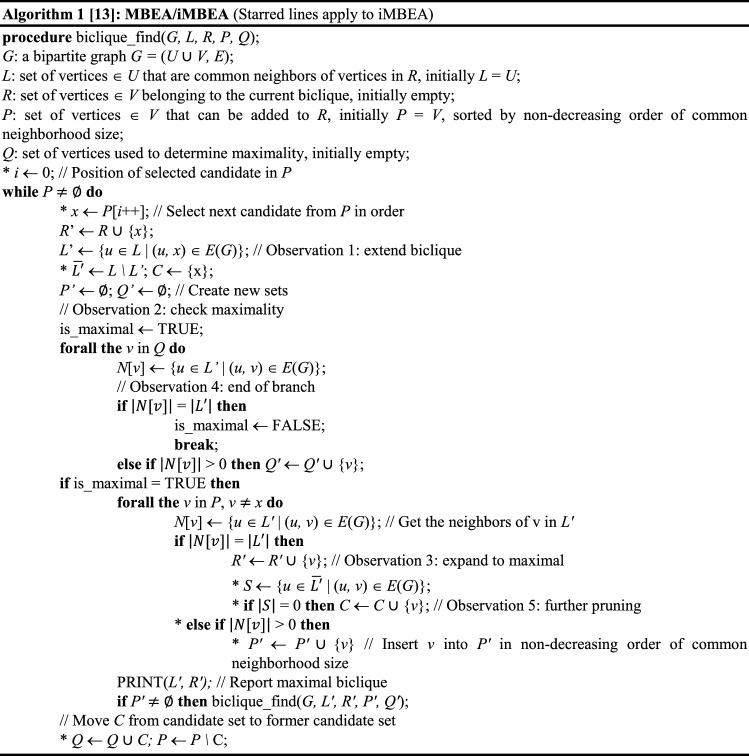


### Application

*Biclique* is invoked in R as follows:

*bicliques *=* bi.clique(filename, left_least, right_least, version, filetype)*

This function generates a list of bicliques, which in the above example are assigned to the *bicliques* variable. The *filename* argument is the name of the input file. Using “left” to denote the first partite set and “right” to denote the second, the *left_least* and *right_least* arguments specify the minimum number of vertices required from each respective partite set in order for a maximal biclique to be reported. The *version* argument specifies whether to use MBEA or iMBEA.

The *filetype* argument can be a little more complicated. It specifies the input file format, which must be either an edge list (0) or a binary matrix (1). The default value is edge list. Such a list is tab-separated, with the first line declaring the number of vertices in each partite set, followed by the number of edges in the graph. Each subsequent line contains a pair of text labels for an edge, with the edge’s left endpoint listed first and its right endpoint second. The binary matrix format is also tab-separated. Example input files are provided with the package.

**Fig. 1:**
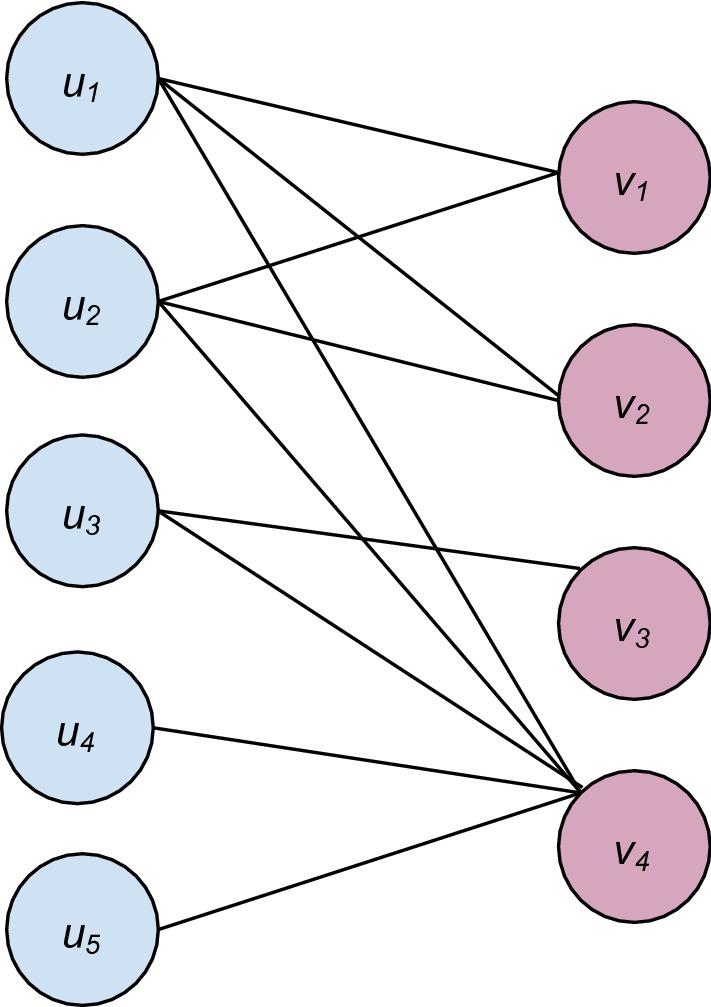
A sample bipartite graph

A sample bipartite graph is depicted in Fig. 1, where vertices *u*_1_, *u*_2_, *u*_3_, *u*_4_ and *u*_5_ are in the left partite set, while *v*_1_, *v*_2_, *v*_3_ and *v*_4_ are in the right. This graph is encoded as graph.el, shown in Table 1.

**Table 1:** The encoding of graph.el, stored in edge list format

5	4	10
u1	v1	
u1	v2	
u1	v4	
u2	v1	
u2	v2	
u2	v4	
u3	v3	
u3	v4	
u4	v4	
u5	v4	

The use of *bi.clique* is exemplified in Sample invocation 1, where *graph.el* denotes the sample graph just illustrated and encoded. Since neither *left_least* nor *right_least* is specified, all maximal bicliques with at least one edge will be reported. Similarly, since no v*ersion* argument is declared, iMBEA will be invoked by default. And since no *filetype* argument is provided, *graph.el* is assumed to be in edge list format. Summary information returned by *bi.clique* comprises a listing of the input’s biclique distribution, its total number of bicliques, and its vertex- and edge-maximum biclique sizes.
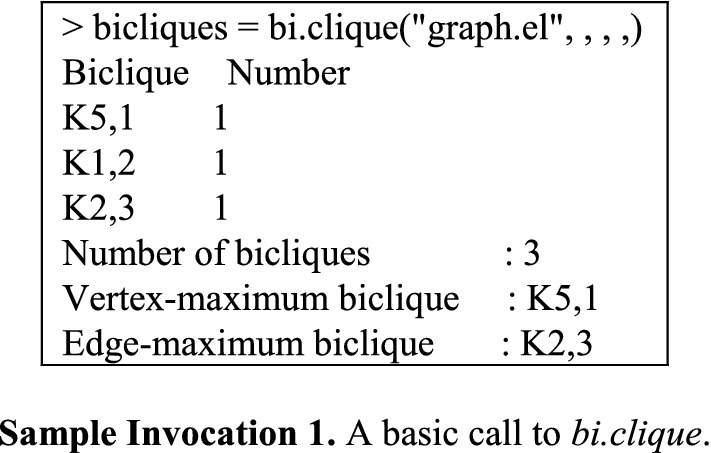


*Biclique* is available on CRAN at https://cran.r-project.org/web/packages/biclique/index.html. Included is an R-style reference manual with detailed descriptions of all arguments and options. This stable, CRAN-ready version can be installed in R with the command *install.packages(“biclique”)*. The latest version of *Biclique* can be obtained via *devtools::install_github(“YupingLu/biclique”)*. Questions or bugs can be submitted to the GitHub webpage. Included in the package are several example bipartite graphs, most of which we obtained from the Koblenz Network Connection [15].

### Tests

All tests were conducted on a Dell server with an Intel Xeon E3-1220 v5 3.0 GHz processor under the Red Hat Enterprise Linux 7 operating system, with 16 GB DDR4 SDRAM, using. R 3.4.2. C code compiled with gcc 4.8.5. Eight bipartite graphs obtained from [15] were studied. As shown in Table 2, timings on them ranged from 0.005 s to 21.094 s. These tests were not meant to be comprehensive, but instead merely to demonstrate that this software can handle affiliation graphs, authorship graphs, interaction graphs and others in addition to the various biological and random graphs tested in [13].

**Table 2:** Timings on eight sample bipartite graphs

Graph	Left	Right	Edges	Bicliques	Vertex-max	Edge-max	Timings
S. African companies	6	5	13	8	K4, 1	K3, 2	0.005
Southern women 2	5	5	14	12	K3, 1	K2, 2	0.005
Southern women 1	18	14	89	63	K14, 1	K5, 4	0.007
Club membership	25	15	95	60	K21, 1	K11, 2	0.006
Corporate leadership	20	24	99	66	K12, 1	K9, 2	0.007
American revolution	136	5	160	14	K59,1	K59,1	0.006
Crime	829	551	1476	620	K1,25	K1,25	0.035
arXiv cond-mat	16,726	22,015	58,595	21,905	K1,116	K1,116	21.094

### Conclusions

*Biclique* provides convenient access, through R, to cutting-edge algorithms for maximal biclique enumeration in bipartite graphs. It provides users with a means to extract relationships between pairs of heterogeneous entities, without a need to worry about implementations of complex codes such as MBEA/iMBEA. Biclique also produces extremal information, including the sizes of vertex-maximum and edge-maximum bicliques. *Biclique* has been tested on a variety of graphs, and is available on both CRAN and GitHub.

### Availability and requirements

Project name: *Biclique*. Project home page: https://github.com/YupingLu/biclique. Operating system(s): Platform independent. Programming language: R. Other requirements: R version 3.4.0 or later is recommended. License: GNU General Public License version 2.0 (GPL-2). Any restrictions to use by non-academics: None.

## Limitations

Biclique enumeration can be output bound. The number of bicliques in large, dense graphs can exceed machine memory limitations.

## Data Availability

Data used in this study are available at the Koblenz Network Collection (http://konect.uni-koblenz.de/).

## Abbreviations

MBEA: Maximal biclique enumeration algorithm
iMBEA: Improved maximal biclique enumeration algorithm
